# The healthcare response to human trafficking: A need for globally harmonized ICD codes

**DOI:** 10.1371/journal.pmed.1002799

**Published:** 2019-05-02

**Authors:** Jordan Greenbaum, Hanni Stoklosa

**Affiliations:** 1 International Centre for Missing and Exploited Children, Alexandria, Virginia, United States of America; 2 Stephanie V. Blank Center for Safe and Healthy Children, Children’s Healthcare of Atlanta, Atlanta, Georgia, United States of America; 3 HEAL Trafficking (Health, Education, Advocacy, Linkage), Los Angeles, California, United States of America; 4 Department of Emergency Medicine, Department of Medicine, Brigham & Women’s Hospital, Harvard Medical School, Boston, Massachusetts, United States of America

## Abstract

In a Perspective, Jordan Greenbaum and Hanni Stoklosa make the case for inclusion of codes for human trafficking in international diagnosis classification systems.

In June 2018, the United States Centers for Disease Control and Prevention added diagnostic codes for forced labor and sexual exploitation to the revised International Classification of Diseases version 10-U.S. (ICD-10-US), a coding system that is applicable only to the US [1]. The global ICD system, created by the World Health Organization (WHO), provides an internationally harmonized language that allows health professionals to share health information across the world. This coding system is critical to monitoring disease incidence and recurrence, determining short- and long-term adverse effects, assessing treatment modalities, and estimating cost of care. In the new ICD version 11 coding system (ICD-11) released by WHO in 2018 [2], various types of child maltreatment are recognized, as well as sexual assault and intimate partner violence and even the rare event of “being hit by a spacecraft.” However, there are no codes for sex or labor trafficking/exploitation. A healthcare provider (HCP) practicing outside of the US must use the generic codes of “sexual abuse” or “sexual assault” in cases of sexual exploitation and has no explicit ability to code for labor exploitation. This is problematic on two levels. First, not all trafficking involves sexual violence. Second, critical information is lost or inaccessible because it is buried within general data on sexual abuse and assault—conditions that do not share many of the characteristics of labor and sex trafficking.

A proposal for new ICD-11 codes on human trafficking (HT) was submitted to WHO in 2014 by the International Centre for Missing and Exploited Children (ICMEC); it was supported by multiple organizations worldwide. The proposal was rejected with no explanation from WHO. We are calling on WHO to include HT among the ICD-11 codes, which will take effect in 2022. Inclusion of diagnostic codes at WHO level is critical to harmonizing international public health efforts to end trafficking.

## Trafficking is a health issue

According to 2016 global estimates, 40.3 million people were victims of modern slavery, including HT [3]. HCPs are only recently recognizing HT as a major public health problem. Trafficked persons may experience myriad adverse health sequelae [4–6], including traumatic injury from work-related accidents and sexual and physical assault, sexually transmitted and other infections, chronic untreated medical conditions, pregnancy and related complications, malnutrition, complications of substance use disorders, post-traumatic stress disorder, major depression, and suicidality. These adverse effects have been demonstrated in survivors of HT from diverse geographic regions, reflecting the global impact of such exploitation. At present, the incidence of the sequelae for each type of exploitation, the risk factors for sequelae, and the cost of treatments are unknown. Access to this information would inform early screening and intervention strategies that could help improve physical and mental health outcomes and reduce the global healthcare burden. For example, recognizing the high risk of suicidality among sex-trafficked youth [7] may drive prompt assessment procedures that prevent fatal self-harm. There is scant anecdotal data on the relationships between the types of exploitation or how sequelae may vary based on the relationship of the trafficked person with the exploiter. For example, risk of substance use may be increased in those who experience combined labor and sexual exploitation or who are exploited by a trusted family member rather than a stranger.

Studies indicate that many trafficked persons seek medical care [8–10]. Accordingly, healthcare professionals have a role in preventing HT by identifying those at risk and offering services to address vulnerabilities. UNICEF, as well as the US Institute of Medicine, has emphasized the need for systematic research to guide public health efforts in identifying and serving survivors [11,12].

## Unique opportunity: ICD-11 codes

Because HCPs have direct contact with survivors of sexual and labor exploitation, it is critical that health systems track the data they gather in a way that allows scientific analysis and informs global health and prevention initiatives.

With specific ICD codes for HT in ICD-11, critical gaps in knowledge may be addressed. Codes will support research on risk and resilience factors, monitor trends in incidence of health adversities associated with HT, support development of treatment and other services, and drive the creation of new prevention strategies. For example, currently little is understood about risk factors for labor trafficking; data gleaned from ICD-11 codes could help identify those at highest risk of labor trafficking, guide creation of supportive interventions, and ultimately prevent high-risk patients from being exploited.

## Mitigating unintended consequences

Adoption of ICD codes must be done with consideration of potential unintended consequences. Trafficked persons may take umbrage at being labeled as a victim of HT, for example. HCPs may be reluctant to use the HT codes for fear of wrongly labeling a patient. In countries in which ICD codes are prominent in the medical record, cultural stigma attached to commercial sex may lead to bias and discrimination against patients by medical staff or retribution by traffickers who gain access to these documents.

To minimize unintended consequences, HCPs and administrators may learn from strategies developed in the fields of intimate partner violence and child maltreatment. Health systems should investigate methods of making ICD codes discreet within the medical records and make a concentrated effort to educate HCPs about use of the codes (the difference between “suspected” and “confirmed” HT, for example) and how to amend outdated or inaccurate codes.

## ICD 11 codes will help track progress toward Sustainable Development Goal 8.7

While we applaud the US for adding HT diagnostic codes to their ICD-10 revisions and encourage other countries to revise their ICD-10 systems, this strategy creates the potential of variations in country-specific codes and nonstandardized data collection. Differing national coding criteria, definitions, and code subcategories will hamper our ability to conduct global-level analysis of trends in prevalence, monitor health impacts, and evaluate treatment modalities across cultures. Instead, the universal use of a single set of ICD-11 codes offers a unique opportunity for the global health community to track progress toward Sustainable Development Goal (SDG) 8.7, which involves the eradication of HT. We urge WHO to adopt diagnostic codes for HT in ICD-11 promptly.

## Abbreviations

HCP: healthcare provider
HT: human trafficking
ICD-10-US: International Classification of Diseases, version 10-United States
ICD-11: International Classification of Diseases, version 11
ICMEC: International Centre for Missing and Exploited Children
SDG: Sustainable Development Goal
WHO: World Health Organization
